# Detection of QTL controlling digestive efficiency and anatomy of the digestive tract in chicken fed a wheat-based diet

**DOI:** 10.1186/1297-9686-46-25

**Published:** 2014-04-03

**Authors:** Thanh-Son Tran, Agnès Narcy, Bernard Carré, Irène Gabriel, Nicole Rideau, Hélène Gilbert, Olivier Demeure, Bertrand Bed’Hom, Céline Chantry-Darmon, Marie-Yvonne Boscher, Denis Bastianelli, Nadine Sellier, Marie Chabault, Fanny Calenge, Elisabeth Le Bihan-Duval, Catherine Beaumont, Sandrine Mignon-Grasteau

**Affiliations:** 1 INRA, UR83 Recherches Avicoles, F-37380 Nouzilly, France; 2 INRA, UMR444 LGC, Chemin de Borde Rouge, BP 52627, F-31326 Castanet Tolosan cedex, France; 3 INRA, UMR1348 PEGASE, Domaine de la Prise, F-35590 Saint Gilles, France; 4 Agrocampus Ouest, UMR1348 PEGASE, F-35042 Rennes, France; 5 INRA, UMR1313 GABI, Domaine de Vilvert, F-78352 Jouy en Josas cedex, France; 6 INRA, LABOGENA, Domaine de Vilvert, F-78352 Jouy en Josas cedex, France; 7 CIRAD, UMR Selmet, F-34398 Monpellier cedex 5, France; 8 INRA, UE1295 PEAT, F-37380 Nouzilly, France

## Abstract

**Background:**

Improving digestive efficiency is a major goal in poultry production, to reduce production costs, make possible the use of alternative feedstuffs and decrease the volume of manure produced. Since measuring digestive efficiency is difficult, identifying molecular markers associated with genes controlling this trait would be a valuable tool for selection. Detection of QTL (quantitative trait loci) was undertaken on 820 meat-type chickens in a F2 cross between D- and D+ lines divergently selected on low or high AMEn (apparent metabolizable energy value of diet corrected to 0 nitrogen balance) measured at three weeks in animals fed a low-quality diet. Birds were measured for 13 traits characterizing digestive efficiency (AMEn, coefficients of digestive utilization of starch, lipids, proteins and dry matter (CDUS, CDUL, CDUP, CDUDM)), anatomy of the digestive tract (relative weights of the proventriculus, gizzard and intestine and proventriculus plus gizzard (RPW, RGW, RIW, RPGW), relative length and density of the intestine (RIL, ID), ratio of proventriculus and gizzard to intestine weight (PG/I); and body weight at 23 days of age. Animals were genotyped for 6000 SNPs (single nucleotide polymorphisms) distributed on 28 autosomes, the Z chromosome and one unassigned linkage group.

**Results:**

Nine QTL for digestive efficiency traits, 11 QTL for anatomy-related traits and two QTL for body weight at 23 days of age were detected. On chromosome 20, two significant QTL at the genome level co-localized for CDUS and CDUDM, i.e. two traits that are highly correlated genetically. Moreover, on chromosome 16, chromosome-wide QTL for AMEn, CDUS, CDUDM and CDUP, on chromosomes 23 and 26, chromosome-wide QTL for CDUS, on chromosomes 16 and 26, co-localized QTL for digestive efficiency and the ratio of intestine length to body weight and on chromosome 27 a chromosome-wide QTL for CDUDM were identified.

**Conclusions:**

This study identified several regions of the chicken genome involved in the control of digestive efficiency. Further studies are necessary to identify the underlying genes and to validate these in commercial populations and breeding environments.

## Background

Feed represents the major cost of production of meat-type chickens i.e. between 55 and 65% depending on the production type [1], and this cost has increased regularly in the last years. For example, in France, the mean cost of poultry feed increased by 34% between 2005 and today. This trend will probably continue because to meet the needs of a rising global human population, it will be necessary to increase crop production both for animal and human consumption and, among others, poultry meat production. Increasing the use of alternative feedstuffs would be one way of reducing the competition between human and animal consumption. However, many of the alternative feedstuffs have a relatively low nutritional value, which results in lower production performance and increased animal manure production. One possibility to tackle this problem is to select birds for improved digestive efficiency. In previous studies, laying hens and meat-type chickens have been selected on traits such as feed conversion ratio (FCR) and residual feed intake (RFI) but the animals were mainly fed with high-quality and easy-to-digest diets [2-5]. Recently, it was shown that genetic selection on digestive efficiency resulted in improved feed efficiency [6,7], with heritability estimates of the digestibility of energy, proteins, lipids and starch ranging from 0.33 to 0.47. These studies were performed on D+ and D- lines that had been divergently selected for high and low digestive efficiency, respectively, and that were measured at three weeks of age after being fed a difficult-to-digest diet. This diet included a high proportion of wheat from the Rialto cultivar, which has been shown to have a poor digestibility [8]. Digestive efficiency was assessed through the apparent metabolizable energy value of the diet corrected to 0 nitrogen retention (AMEn). After eight generations of selection, the AMEn value of the Rialto wheat diet was found to be 33% higher in D+ than in D- birds [9]. When birds were fed a corn diet that was easier to digest, the differences between D+ and D- lines were much smaller but still significant for AMEn and coefficients of digestive utilization, with values 1 to 8% higher in the D+ than in the D- line [10]. These differences in digestive efficiency were associated with changes in the relative sizes of the organs of the digestive tract, i.e. smaller intestines and heavier gizzards in the D+ than in the D- line, as well as changes in gastrointestinal motility and transit time (much faster in the D- than in the D+ line) [11,12]. Non-invasive measurement of digestive efficiency on a large number of animals involves measuring fecal digestibility instead of ileal digestibility, which is time-consuming and requires that animals are kept in cages, *i.e.* in conditions that differ from the current rearing practices. Thus, identification of genetic markers that are involved in the variability of this type of trait would provide valuable tools for marker-assisted selection. Despite the importance of feed efficiency for poultry production, very few QTL (quantitative trait loci) controlling these traits have been detected to date and none were involved in digestive efficiency. In fact, among the 3919 QTL detected so far in poultry (laying hens and broilers), only 26 are related to feed efficiency (http://www.animalgenome.org/cgi-bin/QTLdb*, 02/12/2013*). Furthermore, none of the seven studies reported describe the composition of the diet used or include measurements of digestibility [13-18], although it has been shown that the genetic determinism and level of inheritance of digestive efficiency are closely linked to diet composition [19].

Thus, our aim was to identify QTL controlling digestive efficiency and anatomy of the digestive tract in an F2 cross between the divergent D+ and D- chicken lines fed a suboptimal wheat-based diet.

## Methods

### Animals

The experiment was conducted according to the guidelines of the French Ministry of Agriculture for Animal Research. Chickens from the D+ and D- lines that have been divergently selected on high or low AMEn, respectively [6], were crossed at generation 8 to produce an F2 design. The F2 generation consisted of 820 animals originating from six sires and 60 F1 dams (30 from the cross between D+ males and D- females and 30 from the cross between D- males and D+ females). Five batches of chicks were produced between January and June 2010.

From hatching to 10 days of age, birds were reared in one group on the floor, and then were transferred into individual cages. Throughout the experiment, birds were fed a diet similar to that used during the selection experiment, which contained 55% Rialto wheat (Table 1), except that clinacox replaced robenidine, an anticoccidial drug that has a limited effect on the development of intestinal microbiota [20,21].

**Table 1:** Diet composition

**Ingredients**	**Content (%)**
Corn	6.04
Rialto^1^ wheat	52.53
Soybean oil	6.00
Soybean meal 48	28.40
Corn gluten 60	3.10
Calcium carbonate	1.34
Dicalcium phosphate	1.58
Sodium chloride	0.30
Mineral and vitamin mix^2^	0.35
DL Methionine	0.12
L-Lysine 78	0.22
Clinacox^3^	0.02
**Characteristics**^**4 **^**(calculated)**	
AMEn (kcal.kg^-1^)	3110
Crude proteins (%)	21.1
Lysine (%)	1.16
Methionine + Cystine (%)	0.83
Calcium (%)	1.11
Total phosphorus (%)	0.66
Non-phytate phosphorus (%)	0.42

^1^Wheat was from the Rialto cultivar characterized as “medium hard”, and by a high viscosity of water extract.

^2^Supplied per kilogram of diet and composed of 0.5 mg Co, 16 mg Cu, 47 mg Fe, 1.6 mg I, 65 mg Mn, 0.2 mg Se, 72 mg Zn, 12 000 IU retinyl acetate, 3440 IU cholecalciferol, 80 mg dl-α tocopheryl acetate, 4 mg thiamine, 6.4 mg riboflavin, 20 mg calcium pantothenate, 0.02 mg vitamin B12, 4 mg menadione, 5.6 mg pyridoxine hydrochloride, 0.4 mg folic acid, 0.24 mg biotin, 80 mg niacin, 440 mg choline, 40 mg antioxidant; ^3^Coccidiostat; ^4^Calculated [22].

### Phenotype measurements

For all F2 birds, AMEn values of the diet and coefficients of digestive utilization of dry matter (CDUDM), starch (CDUS), proteins (CDUP) and lipids (CDUL) were individually measured between 17 and 20 days of age using a method based on total excreta collection, as described by Bourdillon et *al*. [23]. The age of 17 to 20 days was chosen because it is the median age of a broilers' rearing cycle. Gross energy, lipid, starch, and protein contents of individual freeze-dried excreta were measured for all birds using the Near Infrared Spectroscopy procedure (NIRS, Foss NIRSystems, Inc., Silver Spring, MD) described by Bastianelli et *al*. [24] with calibration data derived from the chemical analysis of 38 excreta samples.

At 23 days of age, birds were weighed (BW23) and slaughtered, and the gizzard, proventriculus and small intestine were removed, emptied and weighed. Organ weights relative to body weight, expressed as percentages, were designated RGW, RPW, RPGW and RIW for the gizzard, proventriculus, gizzard plus proventriculus, i.e. the upper part of the digestive tract and intestine, respectively. Intestine length relative to body weight (RIL, in cm.g^-1^), intestine density calculated as the ratio of intestine weight to intestine length (ID, in g.cm^-1^), and the proventriculus plus gizzard to intestine weight ratio (PG/I, g.g^-1^) were also calculated. Elementary statistics for these traits are in Table 2.

**Table 2:** Elementary statistics and heritability of the traits related to digestive efficiency and anatomy of digestive tract estimated in F2

**Trait**^**1**^	**Mean**	**Standard deviation**	**h**^**2**^
AMEn (kcal.kg^-1^ DM)	3287	234.00	0.36
CDUDM (%)	70.10	4.82	0.29
CDUS (%)	95.96	4.84	0.33
CDUL (%)	76.86	12.28	0.29
CDUP (%)	81.71	3.89	0.40
RPW (%)	0.80	0.40	0.31
RGW (%)	2.10	0.70	0.64
RPGW (%)	2.83	0.53	0.21
RIW (%)	5.36	0.70	0.20
RIL (cm.g^-1^)	0.26	0.03	0.37
ID (g.cm^-1^)	0.21	0.03	0.30
PG/I (g.g^-1^)	0.54	0.18	0.44
BW23 (g)	451.46	58.50	0.23

^1^AMEn: metabolisable energy value of diet, corrected to 0 nitrogen retention; CDUDM: coefficient of digestive utilization of dry matter; CDUS: coefficient of digestive utilization of starch; CDUL: coefficient of digestive utilization of lipids; CDUP: coefficient of digestive utilization of proteins; RGW: relative gizzard weight to body weight at 23 days of age; RPW: proventriculus weight relative to body weight at 23 days of age; RIW: intestine weight relative to body weight at 23 days of age; RIL: intestine length relative to body weight at 23 days of age; ID: intestinal density; RPGW: proventriculus plus gizzard weight relative to body weight at 23 days of age; PG/I: ratio of gizzard and proventriculus weights to intestine weight at 23 days of age; BW23: body weight at 23 days of age.

### Markers and genotyping

To optimize marker informativity in the experimental cross, a sample of six F0 and six F1 males was genotyped with 57 636 SNPs using the Illumina Infinium chicken SNP array. The 6000 most informative SNPs were selected using the MarkerSet software [25] and were evenly distributed across 28 autosomes, one unassigned linkage group (LGE22C19) and the Z chromosome. They were used to genotype all F0 and F1 individuals and F2 progeny with a dedicated Illumina Infinium custom array.

Low quality SNPs (11.1% with a call rate less than 0.99), SNPs that deviated from the Hardy-Weinberg equilibrium within families (25%) and SNPs that led to inconsistent genotyping relative to pedigree (2.5%) or genetic map (34.3%) information or to both genetic map information and deviations from Hardy-Weinberg rules (27.1%) were discarded from the analysis in order to reduce the risk of erroneous results. Finally, 3379 markers remained (Table 3). The genetic map was deduced from the physical positions of the SNPs and from the genetic consensus reference map published by Groenen et *al*. [26]. This set of markers covered 3099.1 cM.

**Table 3:** Distribution of SNPs used for genotyping and in the final analysis

**Chromosome**	**Initial number of SNPs**	**Final number of SNPs used in the analysis**	**Map length (cM)**^**1**^
1	930	548	483.9
2	710	410	312.4
3	530	268	268.7
4	431	116	202.4
5	286	134	158.3
6	167	89	110.4
7	173	106	113.1
8	133	77	91.6
9	239	120	89.1
10	208	114	89.7
11	211	140	69.2
12	195	116	73.9
13	182	110	58.9
14	159	101	67.5
15	130	71	55.2
16	4	3	0
17	108	60	53.4
18	109	56	52.2
19	96	51	52.6
20	141	76	52.2
21	94	58	52.7
22	50	35	58.9
23	75	42	45.2
24	85	47	47.6
25	21	15	57.4
26	71	60	46.9
27	60	19	52.6
28	56	43	51.6
LGE22C19	12	3	53.2
Z	326	291	231.5

^1^Map length (in cM) of sex-averaged maps according to Groenen et *al*. [26].

### QTL analysis

QTL detection was carried out with the QTLMap software [27] using a half-sib model [28,29] with interval mapping based on maximum likelihood estimations [30]. This model does not make assumptions on the number of QTL alleles segregating in the design. The traits were analyzed separately. Based on a preliminary analysis of variance, some fixed effects were included in the model such as batch (all traits, four levels), sex (CDUS, CDUP, BW, RGW, RPW, RPGW, RIW, RIL, PG/I, two levels), rearing cell (CDUP, three levels), cage row (AMEn, CDUDM, CDUL, CDUP, BW, RGW, RPW, RPGW, RIW, RIL, three levels), slaughter per half-day (RGW, RPGW, PG/I, ID, two levels), person in charge of cutting intestinal segments at slaughter (RGW, RPW, RPGW, RIW, RIL, PG/I, ID, seven levels). QTL analyses were performed by comparing the hypothesis of one QTL (H1) *versus* no QTL (H0) to test the segregation of a QTL on each linkage group.

For each trait, on each chromosome, the significance threshold at the chromosome-wide level was calculated from the results of 1000 simulations of performance under the null hypothesis, with a trait heritability estimated on our design (Table 2). For the most significant QTL, 20 000 simulations were made to derive the genome-wide *p*-value (P_G_) from the chromosome-wide *p*-value (P_C_) using an approximate Bonferroni correction:

$$P_{G}=1-{\left(1-P_{C}\right)}^{1/r},$$

where r was the ratio between length of a specific chromosome and length of the genome as considered for QTL detection in Tilquin et *al.*[31]. Confidence intervals for QTL (95%) were estimated using the LOD drop-off method as proposed by Lander and Botstein [30].

The significance of the QTL effects within each sire family was tested using a Student test, by assuming an equal distribution of the QTL alleles in the progeny. A QTL effect was retained as significant for Student test *p*-values lower than 0.05, and the corresponding sire families were assumed to segregate for this QTL. These familial substitution effects were estimated in families in which significant QTL segregated.

To compute the power of the analysis, 1000 simulations of phenotypes usnder the hypothesis of one QTL for which all sires were heterozygous with an effect of 0.20 phenotypic standard deviation were carried out. The proportion of simulations with a maximum likelihood ratio test larger than the 5% chromosome-wide empirical threshold was computed as the power of the analysis.

## Results

The power of detection of our design did not vary significantly between chromosomes and was always higher than 92%, which means that with this design, it was possible to detect a QTL in more than 92% of cases.

**Figure 1:**
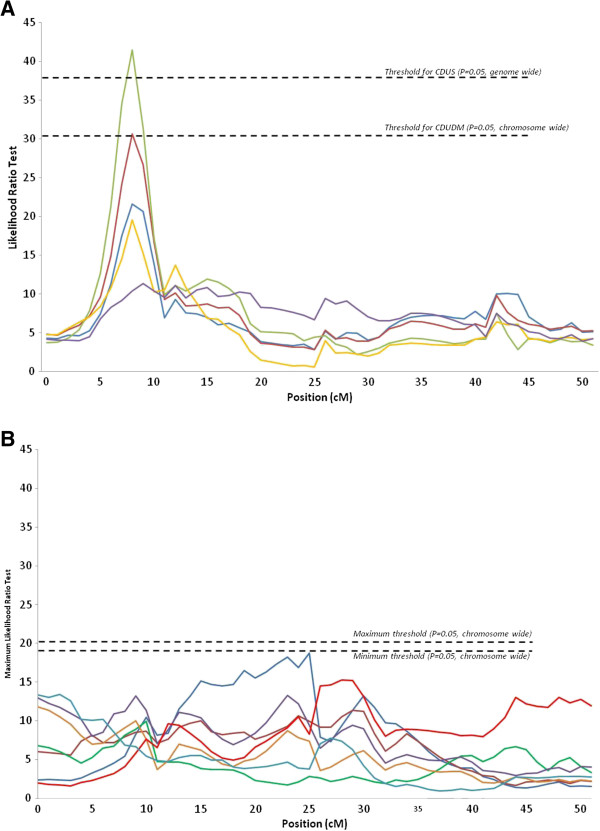
**Curves for the maximum likelihood ratio tests on chromosome 20. (A)** digestibility traits (AMEn in blue, CDUDM in red, CDUS in green, CDUP in yellow, CDUL in purple). **(B)** anatomy of the digestive tract (RGW in dark blue, RPW in dark red, RIW in green, RPGW in purple, PG/I in gold, RIL in light red, ID in light blue).

**Figure 2:**
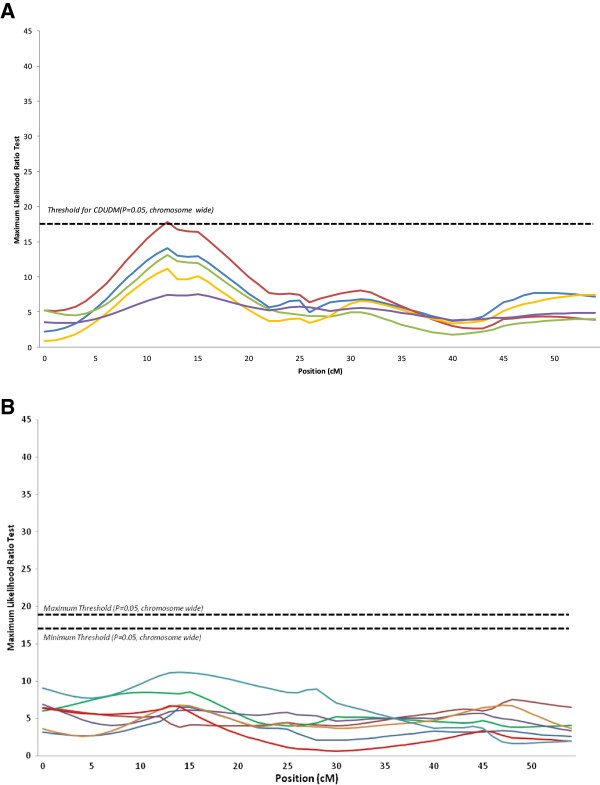
**Curves for the maximum likelihood ratio tests on chromosome 27. (A)** digestibility traits (AMEn in blue, CDUDM in red, CDUS in green, CDUP in yellow, CDUL in purple). **(B)** anatomy of the digestive tract (RGW in dark blue, RPW in dark red, RIW in green, RPGW in purple, PG/I in gold, RIL in light red, ID in light blue).

Nine QTL were detected for digestive efficiency (Table 4), 11 for anatomy of the digestive tract (Table 5) and two for body weight at 23 days of age (Table 5). Most QTL for digestive efficiency were identified for CDUDM and CDUS, two traits which are strongly correlated [32,33]. Two genome-wide QTL for CDUS and CDUDM were observed at the same position on chromosome 20 (Figure 1A). Similarly, four chromosome-wide QTL were found in the same region at 0 cM on chromosome 16 for AMEn, CUDS, CDUDM and CUDP. On chromosome 27, a chromosome-wide QTL for coefficient of digestive utilization of dry matter was detected. It should be noted that for QTL identified on chromosomes 20 and 27, the shape of the likelihood ratio test (LRT) curve was the same for all digestive efficiency traits (Figures 1A and 2A) although the LRT did not reach significance for all of them.

**Table 4:** QTL detected for digestive efficiency

**Chromosome**	**Trait**^**1**^	**Position (cM)**	**CI**^**2**^	**LRT value**^**3**^	**Level of significance**		**QTL effect**^**4 **^**(NF**^**5**^**)**
					**Chromosome-wide**	**Genome-wide**	
16	AMEn	0^6^	-	15.43	0.038	>0.20	0.17 (4)
	CDUDM	0^6^	-	16.57	0.029	>0.20	0.18 (4)
	CDUS	0^6^	-	15.01	0.041	>0.20	0.16 (5)
	CDUP	0^6^	-	18.53	0.012	>0.20	0.20 (4)
20	CDUDM	9	8-10	31.45	<0.0001	0.025	0.40 (5)
	CDUS	9	8-10	41.60	<0.0001	<0.0001	0.57 (4)
23	CDUS	30	8-35	19.45	0.049	>0.20	0.22 (3)
26	CDUS	36	31-45	20.05	0.041	>0.20	0.20 (3)
27	CDUDM	12	9-17	18.10	0.046	>0.20	0.20 (3)

^1^AMEn: metabolisable energy value of diet, corrected to 0 nitrogen retention; CDUDM: coefficient of digestive utilization of dry matter; CDUS: coefficient of digestive utilization of starch; CDUL: coefficient of digestive utilization of lipids; CDUP: coefficient of digestive utilization of proteins; ^2^1-LOD-drop off confidence interval (lower and upper boundaries, cM); ^3^Likelihood Ratio Test value; ^4^QTL effect as a proportion of the phenotypic standard deviation of trait; ^5^number of F1 sires families heterozygous for the QTL (P < 0.05, Student test); ^6^QTL located at the telomere.

**Table 5:** QTL detected for anatomy of the digestive tract

**Chromosome**	**Trait**^**1**^	**Position (cM)**	**CI**^**2**^	**LRT value**^**3**^	**Level of significance**		**QTL effect**^**4 **^**(NF**^**5**^**)**
					**Chromosome-wide**	**Genome-wide**	
1	BW23	463	455-465	31.05	0.007	0.034	0.25 (4)
	RPGW	456	453-462	29.63	0.019	0.093	0.20 (4)
6	RPW	84	74-87	20.63	0.048	>0.20	0.23 (3)
8	RIL	3	0-6	20.74	0.025	>0.20	0.20 (4)
	RGW	44	35-49	19.46	0.045	>0.20	0.14 (4)
11	PG/I	17	13-21	19.98	0.034	>0.20	0.20 (4)
12	ID	44	43-45	20.79	0.039	>0.20	0.22 (3)
16	RIL	0^6^	-	15.64	0.030	>0.20	0.25 (2)
18	RIL	45	38-51	20.11	0.035	>0.20	0.19 (5)
21	PG/I	27	13-34	19.60	0.047	>0.20	0.20 (4)
	RPGW	27	14-33	20.12	0.040	>0.20	0.22 (4)
26	BW23	25	21-26	26.83	0.0005	0.106	0.31 (2)
	RIL	37	34-40	23.09	0.010	>0.20	0.27 (2)

^1^RGW: relative gizzard weight to body weight at 23 days of age; RPW: relative proventriculus weight to body weight at 23 days of age; RIL: relative intestine length to body weight at 23 days of age; RPGW: relative gizzard weight and proventriculus weight to body weight at 23 days of age; ID: intestinal density; PG/I: ratio of gizzard and proventriculus weights to intestine weight at 23 days of age; BW23: body weight at 23 days of age; ^2^1-LOD-drop off confidence interval (lower and upper boundaries, in cM); ^3^Likelihood Ratio Test value; ^4^QTL effect as a proportion of the phenotypic standard deviation of trait; ^5^number of F1 sire families heterozygous for the QTL (P < 0.05, Student test); ^6^QTL located at the telomere.

Among the 11 QTL found for anatomy of the digestive tract, four were involved in traits related to the upper part of the tract (i.e. gizzard and proventriculus) on chromosomes 1, 6, 8 and 21, five in traits related to the lower part of the tract (i.e. small intestine) on chromosomes 8, 12, 16, 18, 26, and two in traits related to the ratio between upper and lower parts of the gastrointestinal tract (PG/I) on chromosomes 11 and 21. All these QTL were detected only at the chromosome-wide level. On chromosome 21, a co-localization between a QTL for the relative weight of the upper part of the digestive tract and a QTL for the ratio between upper and lower parts of the gastrointestinal tract was observed.

**Figure 3:**
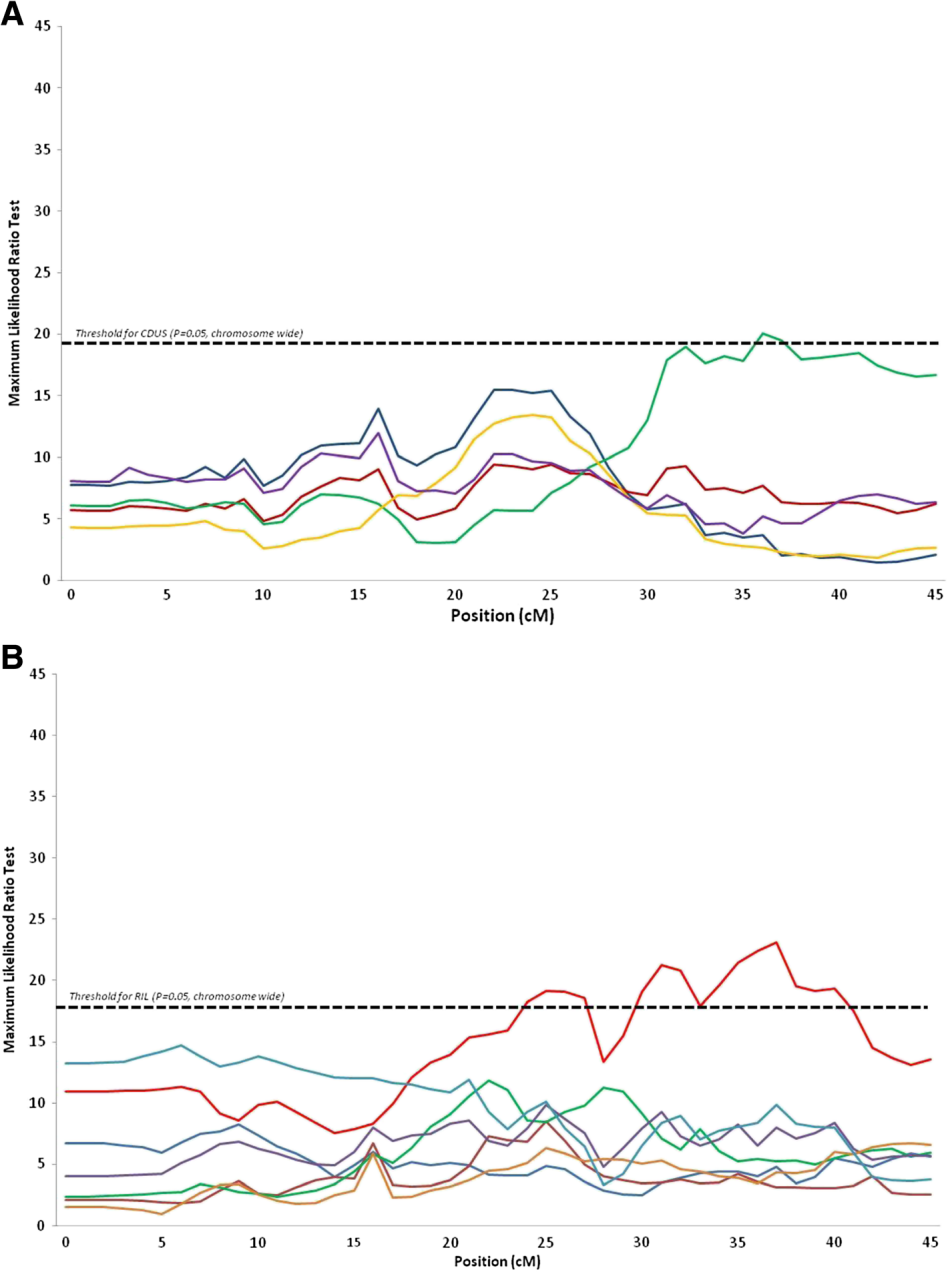
**Curves for the maximum likelihood ratio tests on chromosome 26. (A)** digestibility traits (AMEn in blue, CDUDM in red, CDUS in green, CDUP in yellow, CDUL in purple). **(B)** anatomy of the digestive tract (RGW in dark blue, RPW in dark red, RIW in green, RPGW in purple, PG/I in gold, RIL in light red, ID in light blue).

Co-localization of QTL for digestive efficiency and anatomy traits was found only on chromosomes 16 (AMEn, CDUDM, CDUS, CDUP, RIL) and 26 (CDUS and RIL) (Figures 3A and B).

Overall, the magnitude of the effects of QTL ranged from 0.15 to 0.60 phenotypic standard deviation, with the greatest effect found on chromosome 20 for a genome-wide significant QTL. This QTL was significant in most of the sire families of the design (four and five families depending on the traits), while the effects of the QTL for the other digestive efficiency traits and for body weight were significant in two to five sire families.

The 95% confidence interval for the QTL localizations ranged from 2 cM on chromosome 20 (CDUS and CDUDM) and chromosome 12 (ID) to 27 cM on chromosome 23 (CDUS). The intervals tended to be narrower for the most significant QTL.

## Discussion

This study is the first to highlight the presence of QTL for digestive efficiency in the chicken. Nine QTL involved in digestive efficiency traits, and 11 QTL in anatomy-related traits were found. Indeed, the power of the study was enhanced both by testing the birds on a low-quality diet, which revealed a high variability among the birds, and by studying a cross between divergent lines.

However, it should be noted that except for the QTL on chromosome 20, most of the QTL were only significant at the chromosome level (*P* < 0.05), which is probably due to the fact that they are not fixed in the F0 population in which the D+ and D- lines have alternate alleles: only three or four of the six F1 sire families were found heterozygous at these QTL. This observation is also consistent with the complexity of digestive efficiency traits which are most probably polygenic traits. Although digestive efficiency assessed by AMEn is one of the components of FCR, it includes many physiological processes such as digestive secretions, absorption, motility and neurohumoral coordination, the roles of which also vary with nutrients. In addition, the weak effects of most QTL suggest polygenic control, except for the QTL that are involved in CUDS on chromosome 20 and RIL on chromosome 16, although the latter was significant in only two families.

Since the digestive efficiency of the whole diet depends on the digestive efficiency of each of its nutrients, QTL that control digestive efficiency were found mainly for components of AMEn (mainly CDUS) and not for AMEn itself, in spite of significant and highly positive genetic correlations (0.60 to 0.90). This result was consistent with those of previous QTL studies [13-18,34] that were focused on FCR and that detected more QTL for components of the FCR, such as feed intake, growth and body composition than for FCR itself, i.e. nine QTL were found for feed conversion ratio but 16 for production traits such as growth or egg production and 13 for body composition.

In our study, the largest number of QTL was found for CUDS. Indeed, the major component of the dietary content is starch, which can explain why selection on digestive efficiency affected mostly digestibility of starch. Moreover, digestion of starch probably involves fewer physiological limiting factors than that of other components of the diet, since only a few types of hydrolytic enzymes (α-amylase, maltase, isomaltase) and a very efficient absorption of glucose, the end product, are required. It is generally accepted that the potential for starch hydrolysis in terms of enzyme secretions is very high in chickens [35]. Thus, the low digestive utilization of starch observed in some birds in this study originated probably from disorders of digestive motility [12] or of the coordination between motility and pancreatic secretions. It should be noted that no QTL was detected for the coefficient of digestive utilization of lipids. Although its heritability was similar to that of the other traits, it also depended on an interaction between the bird and its intestinal microbiota, especially on the balance between *Lactobacillus salivarius* and *Escherichia coli* and between *Lactobacillus salivarius* and *Clostridium leptum* (data not shown). This double contribution of both the bird and its intestinal microbiota to the digestibility of lipids can make QTL detection more difficult.

Although the results depended on the nature of the nutrients, we identified two regions that carry QTL for several traits on chromosomes 16 (four QTL) and 20 (two QTLs). All traits related to digestive efficiency showed a peak at these positions but did not reach the significance threshold. This result is in agreement with the strong genetic correlations observed between the different digestive efficiency traits [19]. However, on chromosomes 23 and 27 neither co-localized QTL nor closely localized QTL were observed, which suggests either that the genetic control varies, at least partially, between traits, or that this result is a false negative. These results cannot be attributed to a lack of power of our design, which exceeded 92% for all QTL.

In our study, we did not find any QTL at positions previously reported for feed efficiency traits that were recorded for animals fed a high-quality diet [13-18], except for chromosome 16 for which Ewald et *al*. [18] published a QTL for feed conversion ratio. However, given the poor precision of this localization on chromosome 16, we cannot exclude the possibility that these two QTL are the same. This general discrepancy between QTL for feed efficiency and digestive efficiency is not surprising. Digestive efficiency is one of the components of feed efficiency that also includes growth rate, body composition, feed intake and heat production. Furthermore, these studies were undertaken with animals fed on high-quality diets, which means that the contribution of digestive efficiency to the variation of the FCR was fairly low. Moreover, another difference is that the chicken used in these earlier studies grew considerably faster than the chickens used in our study.

Data for QTL published in the literature and for those from our study were more consistent for traits related to the anatomy of the digestive tract than for digestive efficiency traits. On chromosome 1, we detected a QTL for RPGW at 456 cM, while Gao et *al*. and Nones et *al*. reported overlapping QTL for gizzard weight at a distance of about 50 cM from our QTL but with very large confidence intervals [36,37]. Thus, we cannot completely exclude the possibility that the QTL for gizzard weight and the QTL for RPGW are the same. On chromosome 11, the QTL that we detected for PG/I was within the very large confidence interval (0 to 35 cM) reported for the QTL for intestine length by Gao et *al*. [37]. On chromosome 26, the confidence interval of the QTL for RIL at 37 cM (34–40 cM) did not overlap with the *G0S2* gene but was still quite close to it; the *G0S2* gene was found to affect intestine length [38]. Therefore, the identification of co-localized QTL for gizzard weight and RPGW is not surprising, if one considers that greater intestinal development can be interpreted as an attempt to counterbalance a functional disorder of the gizzard [11].

On chromosome 26, co-localized QTL for CDUS and relative intestine length were detected, which is consistent with the strong phenotypic differences in gastrointestinal tract morphology observed between D+ and D- lines, with a heavier gastric compartment and a lighter small intestine in the D+ than in the D- line [10,11,39] and also with the genetic correlations between digestive efficiency and anatomy traits, in particular weight of the intestine [32]. Identification of the genes that underlie these QTL should make it possible to distinguish between the effect of gene(s) that control both variation in digestive efficiency and gut morphology and a physiological effect of digestive efficiency on gut morphology.

Even if QTL have only moderate effects, selection on these traits may result in a significant reduction in production costs. For instance, the QTL for body weight at 23 days of age on chromosome 1 was responsible for a 2.3% increase in body weight. Extrapolating this difference to the whole production cycle results in an increase in body weight of 46 g per chick at 23 days of age which, when multiplied by the number of chickens per year per laying house (around 120 000), represents an increase of 5520 kg of meat per henhouse and per year. Similarly, increasing starch digestibility by 2.2% (as for the QTL on chromosome 20) could save 4 tons of feed per year per laying house.

## Conclusion

Based on the fact that feeding chickens on a poor diet increased the genetic variability of the traits of interest, and thus the power of QTL detection, it can be assumed that the QTL detected in this study could also be expressed when birds are fed a more digestible diet. Previous studies showed that the extent of the differences between the D+ and D- lines depended on diet composition [40], and that the anatomical differences were not present at hatching [9,10]. Thus, animals reacted to the diets while challenged. However, even if reduced, most differences between these lines remained significant when high-quality diets were used [10,11], which suggests that the QTL detected here are also involved in the variations in digestive efficiency observed with more favorable diets. This is consistent with the high and positive genetic correlations between digestive efficiency traits estimated on wheat and corn diets [19]. Another line of study that should be addressed in the future is the possibility that epigenetic phenomena affect the expression of genes controlling digestive efficiency without changing the positions of the QTL themselves.

## Abbreviations

AMEn: Metabolisable energy value of diet, corrected to 0 nitrogen retention; BW23: Body weight at 23 days of age; CDUDM: Coefficient of digestive utilization of dry matter; CDUS: Coefficient of digestive utilization of starch; CDUL: Coefficient of digestive utilization of lipids; CDUP: Coefficient of digestive utilization of proteins; ID: Intestinal density; PG/I: Ratio of gizzard and proventriculus weights to intestine weight at 23 days of age; RGW: Relative gizzard weight to body weight at 23 days of age; RIL: Relative intestine length to body weight at 23 days of age; RIW: Relative intestine weight to body weight at 23 days of age; RPGW: Relative gizzard weight and proventriculus weight to body weight at 23 days of age; RPW: Relative proventriculus weight to body weight at 23 days of age.

## Competing interests

The authors declare that they have no competing interests.

## Authors’ contributions

All authors contributed to the preparation of the manuscript and to the scientific discussions. TST contributed to the genetic analyses, AN to the conception and design of the study and phenotyping of the digestive tract, BC to the conception and design of the study and phenotyping for the AMEn measurements, IG and ELBD to the conception and design of the study and to phenotyping, NR to phenotyping, HG to the genetic analyses and development of the QTLMap software, OD to the verification of genotypes, BB to the verification of genotypes and markers, CCD and MYB to the genotyping of animals, DB to the measurement of digestive efficiency, NS to the supervision of animal rearing, MC to the reproduction of experimental lines and creation of the F2 cross, CB to the conception of the QTL analysis and SMG to the coordination of the project and the conception and design of the selection of experimental lines. All authors read and approved the final manuscript.
